# Minocycline Attenuates Experimental Subarachnoid Hemorrhage in Rats

**DOI:** 10.1515/biol-2019-0067

**Published:** 2019-12-31

**Authors:** Jingbo Li, Shuda Chen, Jing Fan, Gao Zhang, Reng Ren

**Affiliations:** 1 Neurocritical Care Unit, the Second Affiliated Hospital, Zhejiang University School of Medicine, 88 Jiefang Road, Hangzhou 310009, Zhejiang Province, P.R. China

**Keywords:** subarachnoid hemorrhage, minocycline, microglia, P2X4 receptor, early brain injury

## Abstract

**Backgroud:**

The aim of this study was to evaluate the therapeutic effect of minocycline on treating experimental subarachnoid hemorrhage (SAH) in rats and to explore its possible molecular mechanism.

**Methods:**

SAH was induced in male Sprague-Dawley rats by endovascular perforation. The rats were treated with minocycline (25 mg/kg or 50 mg/kg) or saline at 2 hand 12 h post SAH. Neurological function, cerebral hemorrhage, and edema were scored at 48 h post SAH. Cell death and P2X4 receptor (P2X4R) expression were observed in the prefrontal cortex (PFC).

**Results:**

Treatment with a highdose of minocycline significantly improved the neurological function score, and attenuated cerebral hemorrhage and edema. Low-dose minocycline could reduce hemorrhage, but the effect on neurological deficits and brain edema was not obvious. Minocycline treatment could alleviate neuronal apoptosis in the PFC, which was related to reduced expression of inflammatory cytokines. Immunofluorescence showed that P2X4R on microglia was activated after SAH. Minocycline treatment inhibited P2X4R activation and further suppressed the phosphorylation of downstream p38 MAPK.

**Conclusions:**

Minocycline plays a neuroprotective role by attenuating early brain injury after experimental SAH. The therapeutic mechanism of minocycline may be mediated by the inhibition of P2X4R on microglia.

## 1 Introduction

Subarachnoid hemorrhage (SAH) is a common severe cerebrovascular disease in the neurology department. SAH is usually caused by a rupture of intracranial aneurysms or traumatic factors [1]. In addition to surgical management, medical management is applied to control cerebral and extracerebral complications such as recurrent hemorrhage, vasospasm, cerebral edema, respiratory failure, myocardial dysfunction, etc. It is widely believed that patients can benefit from intensive treatment. However, the prognosis of SAH is usually poor. In addition to the high mortality, survivors often suffer from severe neurological impairment, which brings a heavy socioeconomic burden [2, 3]. The main reason for poor prognosis of SAH is early brain injury (EBI), i.e. a series of primary brain injuries and secondary pathophysiological changes within 72 hours [4]. An early and effective intervention might be a practical way to improve the outcome of SAH patients.

Microglial cells play a principle role in triggering the inflammatory response and phagocytosis of rogue erythrocytes after SAH [5, 6, 7]. After SAH, brain microglia are activated, triggering intracellular inflammation and releasing a large number of inflammatory factors, leading to the destruction of the blood-brain barrier, further aggravating the infiltration of macrophages and brain edema [8]. Minocycline (7-dimethylamino-6-dimethyl-6-deoxytetracycline) is a specific inhibitor of microglia [9]. Due to its unique ability of passing the blood-brain barrier, minocycline has been found to have many neuroprotective activities in several neurodegenerative disorders such as ischemic stroke, traumatic brain injury, Alzheimer’s disease, and Parkinson’s disease [10, 11, 12]. Several studies also suggested that minocycline might have therapeutic potential in experimental SAH rats [13,14]. These previous studies showed that minocycline could inhibit expression of matrix metalloproteinase (MMP)-9 in the hippocampus and reduce the production of reactive oxygen species (ROS) in the cortex. P2X4 receptor (P2X4R) is an ATP-gated cation channel and is dominantly expressed in hyperactive microglia but not in neurons or astrocytes [15]. The current study aims to investigate whether the therapeutic effect of minocycline on SAH involves the regulation of P2X4R.

## 2 Materials and methods

### 2.1 Animals and SAH model

Forty-four male Spraque-Dawley rats (weighing 280-350 g) were purchased from the Shanghai Laboratory Animal Company (Shanghai, China) and used for SAH induction. The animals were raised with enough food and water under controlled temperature and humidity. The rats were randomly divided into 4 groups: sham group (n=8), SAH+saline group (n=12), SAH+low-dose minocycline group (n=12), SAH+high-dose minocycline group (n=12). The SAH model was induced using the endovascular perforation method as reported [16]. General anesthesia was induced with 5% isoflurane and maintained with 3% isoflurane. After exposing the left common carotid artery (CCA), external carotid artery (ECA), and internal carotid artery (ICA), the ECA was ligated and fashioned into a 3-mm stump. A 4-0 nylon suture was advanced into the intracranial ICA through the ECA stump until resistance was felt and pushed 3 mm further to perforate the arterial wall. The suture was then withdrawn and the ICA was reperfused. For the sham group, all the procedures were the same except that the suture was withdrawn without perforating the artery.

**Ethical approval**: The research related to animals use has been complied with all the relevant national regulations and institutional policies for the care and use of animals.

### 2.2 Treatment of minocycline

Minocycline hydrochloride powder was purchased from Sigma-Aldrich (St. Louis, MO, USA) and dissolved in normal saline (NS) to make a solution of 50 mg/mL. The rats were treated with minocycline or 1 mL of saline at 2 h and 12 h post SAH. For the low-dosage group, each rat was intraperitoneally injected with 25 mg/kg of minocycline each time to make a total dose of 50 mg/kg; for the high dosage group, each rat was intraperitoneally injected with 50 mg/kg of minocycline each time to make a total dose of 100 mg/kg. The dose of treatment and the injection time point were referred to previous studies [13, 17].

### 2.3 Neurological function score

Six rats were randomly selected from each group at 48 h post SAH and their neurological function was scored using modified Garcia method [17, 18]. Six parameters, scoring from 0-3, were evaluated by a blinded observer. The higher the total score (ranging from 3 to 18), the better the neurological functions of the animals were.

### 2.4 Cerebral hemorrhage score

After a neurological function test, three rats were randomly selected from each group and sacrificed under deep anesthesia. The brains were removed quickly for observation of cerebral hemorrhage. The severity of hemorrhage was scored by a blinded observer based on the grading system of Sugawara et al. [16]. Six segments of the basal cistern were allotted a score from 0-3 based on the amount of subarachnoid blood clot in each segment. Higher total scores (ranging from 0 to 18) represented more severe bleeding.

### 2.5 Brain water content (BWC) measurement

After observation of hemorrhage severity, the brain samples were tested for BWC as reported [19]. Brains were separated into left and right hemispheres, brainstem, and cerebellum. The specimens were firstly weighed for wet weight and secondly weighed for dry weight after being dried at 100°C for 72 hours in an oven. The percentage of BWC was calculated as (wet weight-dry weight)/wet weight×100%.

### 2.6 Histopathological examination by immunofluorescence and TUNEL

Three randomly selected rats of each group were sacrificed under deep anesthesia and transcardially perfused with ice-cold saline, followed by 4% paraformaldehyde. The brain tissues were fixed in 10% formaldehyde at 4°C overnight and 30% sucrose solution until saturation. The brains were then frozen in optimum cutting temperature compound and cut into 10-μm coronary sections in cryostat (CM3050S, Leica Microsystems, Buffalo, IL, USA). To detect the expression of P2X4R, double fluorescence labeling was performed on brain sections as described [14]. The sections were washed with PBS and blocked with 10% normal goat serum. Subsequently, the sections were incubated with the primary antibodies at 4°C overnight. Afterward, the sections were washed with PBS and incubated with secondary antibodies at room temperature for 2 hours in the dark. The primary antibodies used were mouse anti-Iba-1 (1:100, Abcam Inc., Cambridge, UK) and rabbit anti-P2X4R (1:200, Alomone Labs, Jerusalem, Israel). The secondary antibodies used were FITC-conjugated goat anti-mouse IgG (1:50, Aspen Biotechnology, Wuhan, China) and TRITC-conjugated goat anti rabbit IgG (1:50, Aspen). The sections were washed again and stained with DAPI, then mounted with glycerol. The sections were examined under a fluorescence microscope (IX51, Olympus) and merged by Image-Pro Plus 6.0 software (Olympus, Melville, NY, USA). Apoptosis of neuron cells were detected using TUNEL staining kit (Roche Life Science, Basel, Switzerland) according to the manufacturer’s instruction and examined under a fluorescence microscope.

### 2.7 Western blot

Cytoplasmic protein extracts were prepared from the left hemispheres (perforation side). The brain tissues were homogenized in an RIPA buffer (Boster Biological Technology, Wuhan, China) and centrifuged (1000×*g*) at 4°C for 10 minutes. The protein concentration was determined using a BCA protein assay kit (Boster). Equal amounts of total protein (50 μg/sample) were isolated on 10% SDS-polyacrylamide gel and transblotted onto a nitrocellulose membrane. Membranes were blocked with 5% non-fat milk and incubated with specific primary antibodies at 4°C overnight. The primary antibodies used were: rabbit anti-interleukin (IL)-1β (1:1000, Abcam), rabbit anti-tumor necrosis factor (TNF)-α (1:1000, Abcam), rabbit anti-caspase-3 (1:500, Abcam), rabbit anti-P2X4R (1:500, Alomone Labs), rabbit anti-p38 (1:2000, Abcam), rabbit anti-p38 (phosphor T180+Y182, 1:1000, Abcam). GAPDH was detected as a loading control by using rabbit anti-GAPDH (1:5000, Abcam). The next day, membranes were incubated with the secondary horseradish peroxidase-conjugated goat anti-rabbit IgG (1:2000, Abcam) at room temperature for 1 h after TBST washing. Blot bands were visualized with enhanced chemiluminescence kit (Boster) and band density was quantified by ImageJ software.

### 2.8 Statistical analysis

The data obtained from the above experiments were analyzed by GraphPad Prism5 (San Diego, CA, USA). Quantitative data were expressed as mean±SD. The differences between the two groups were analyzed by t-test. Difference among multiple groups was analyzed by one-way ANOVA followed by Tukey’s post-hoc test. Statistical significance was defined as *P* value <0.05.

## 3 Results

### 3.1 Effect of minocycline on neurologicdys-function, SAH grade, and brain edema

Endovascular perforation will cause postoperative deaths (10% to 50%) [20]. In our study, the mortality rate was 0% in the sham group, 16.7% (2/12) in the SAH+saline group, 16.7% (2/12) in the SAH+low-dose minocycline group, and 25% (3/12) in the SAH+high-dose minocycline group. All the deaths occurred within 24 h post SAH and there was no difference among the mortality rates (*P*=1.00). Those dead rats were excluded and at least six surviving rats remained in the subsequent assessment.

**Figure 1:**
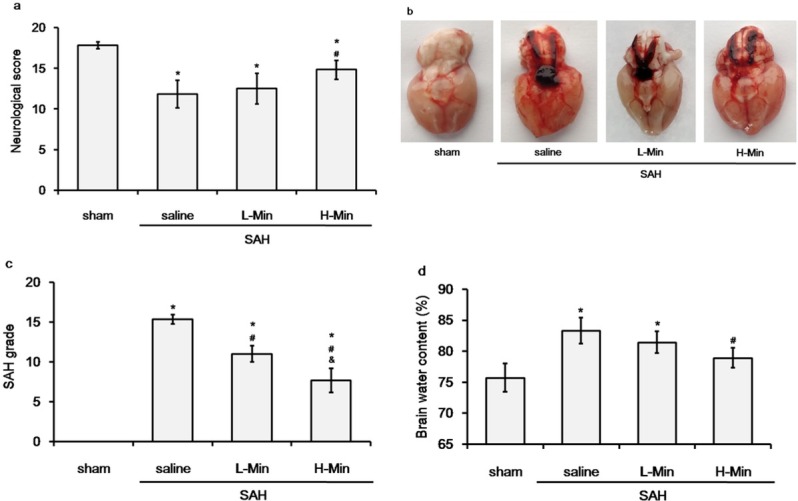
The effect of minocycline on neurological scores, SAH grade, and brain edema at 48 hours post SAH. a. Quantification of neurological scores. b. Typical brains from sham, SAH+saline, SAH+low-dose minocycline (L-Min), and SAH+high-dose minocycline (H-Min) groups. c. SAH grade of different groups. d. Percentage of brain water content (BWC) in different groups. *P<0.05 vs. sham; #*P*<0.05 vs. saline; $*P*<0.05 vs. L-Min.

The neurological function was assessed 48 hours post SAH. As shown in Figure 1a, the Garcia scores were significantly lower in the SAH rats compared to the sham group (11.83±1.72 vs. 17.83±0.41, *P*<0.01). The high-dose of minocycline significantly improved the Garcia scores compared to the saline group (14.83±1.17 vs. 11.83±1.72, *P*=0.027), but failed to fully restore the level of the sham group (14.83±1.17vs. 17.83±0.41, *P*<0.01). However, the neurological deficit was not improved in the SAH rats that received the low-dose minocycline treatment (12.50±1.87 *vs*. 11.83±1.72, *P*=0.535). The difference of neurological deficit was significant among the saline and minocycline groups (*P*=0.014).

The average SAH grade was 15.33±0.58 in the saline group, 11.01±1.01 in the low-dose minocycline group, and 7.67±1.53 in the high-dose minocycline group (Figure 1b&1c). According to the severity of cerebral hemorrhage, minocycline treatment could attenuate the severe hemorrhage to moderate (low-dose) or mild-moderate (high-dose). The difference was significant among the three groups (*P*<0.001).

SAH induction caused severe brain edema at 48 hours post operation. The BWC was significantly increased in the SAH rats treated with saline or low-dose minocycline compared to the sham group (83.32±2.11% vs. 75.70±2.26% and 81.41±1.75% vs. 75.70±2.26%, both *P*<0.05). Low-dose minocycline did not reduce the BWC compared to the sham group (*P*=0.295). Only high-dose minocycline could reduce the BWC to normal level (*P*=0.045 vs. saline and *P*=0.116 vs. sham).

### 3.2 Minocycline inhibits apoptosis of neuron cells and attenuates the release of inflammatory factors

**Figure 2:**
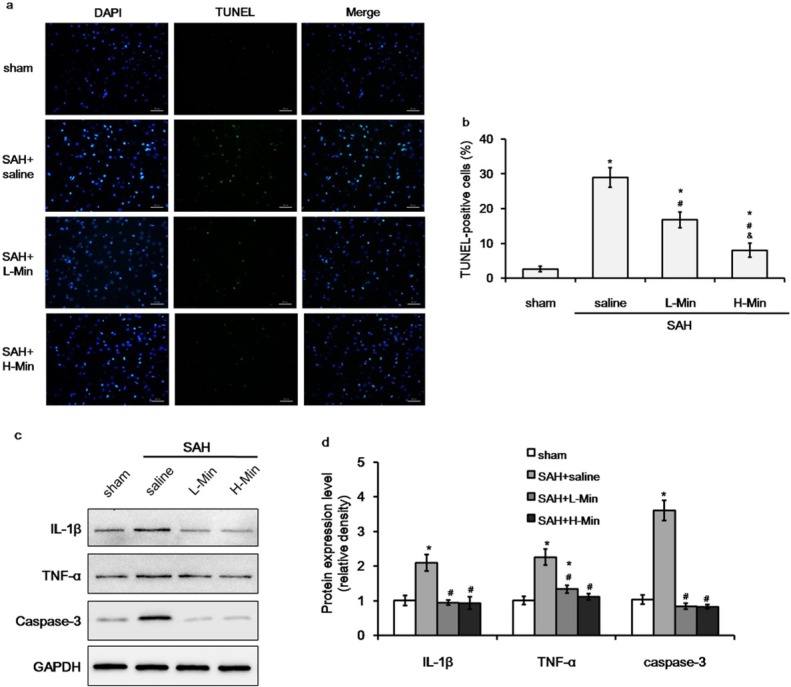
The effect of minocycline on apoptosis of neuron cells and expression of inflammatory factorsat 48 hours post SAH. a. TUNEL staining of neuron cells in the prefrontal cortex region (magnification ×400, scale bar=50 μm). b. Percentage of TUNEL-positive cells in sham, SAH+saline, SAH+low-dose minocycline (L-Min), and SAH+high-dose minocycline (H-Min) groups.c&d. Protein expression of inteleukin (IL)-1β, tumor necrosis factor (TNF)-α, and caspase-3. *P<0.05 vs. sham; #*P*<0.05 vs. saline.

Previous study showed that apoptotic neuron cells markedly increased in the ipsilateral fronto-basal cortex after SAH [21]. Therefore, we chose the prefrontal cortex (PFC) for histopathological observation. TUNEL staining showed that apoptotic neuron cells were rarely detected in the sham group, but neuronal apoptosis was significantly increased after SAH (Figure 2a&2b, *P*<0.01)). The increased expression of inflammatory factor (IL-1β and TNF-α) and apoptotic marker caspase-3 was also observed by Western blot (Figure 2c&2d, all *P*<0.01). Minocycline treatment markedly inhibited apoptosis of neuron cells and attenuated the expression of inflammatory factors and the difference was significant among the groups of saline, low-dose minocycline, and high-dose minocycline (Figure 2, all *P*<0.01).

### 3.3 Minocycline inhibits activation of P2X4R in microglia

**Figure 3:**
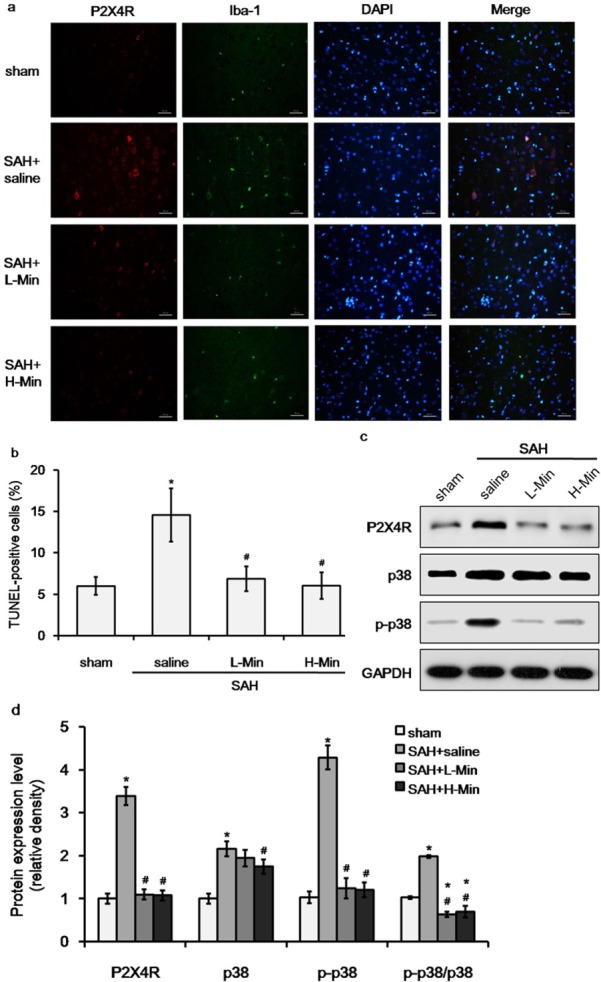
The effect of minocycline on activation of P2X4R and p38at 48 hours post SAH.a. Double fluorescence labeling of P2X4R (red) and Iba-1 (green) in the prefrontal cortex region (magnification ×400, scale bar=50 μm).b. Percentage of P2X4R-positive cells in sham, SAH+saline, SAH+low-dose minocycline (L-Min), and SAH+high-dose minocycline (H-Min) groups.c&d. Protein expression of P2X4R, total p38, p-p38, and p-p38/total p38. *P<0.05 vs. sham; #*P*<0.05 vs. saline.

Next, we investigated whether P2X4R expression was regulated by minocycline. Microglia in the PFC was detected by labeling Iba-1 (green fluorescence) and P2X4R was labeled by red fluorescence. As shown in Figure 3a and 3b, the number of P2X4R-positive cells was significantly increased in the saline group compared with the sham group (*P*=0.012). Minocycline treatment effectively reduced the number of P2X4R-positive cells and the difference was significant among the groups of saline, low-dose minocycline, and high-dose minocycline (Figure 3a&3b, *P*<0.01). Western blot also showed that the protein level of P2X4R was up-regulated after SAH and could be reduced by minocycline (Figure 3c&3d). A previous study reported that the activation of P2X4R could lead to the activation of p38 mitogen activated protein kinase (MAPK) [22]. Therefore, we also detected the expression of p38 and its active form p-p38. Western blot (Figure 3c&3d) showed that the overall expression trend of p38 was consistent with that of P2X4R post SAH. More specifically, the expression of total p38 was not different among the groups of saline, low-dose minocycline, and high-dose minocycline (*P*=0.075); however, minocycline significantly inhibited phosphorylation of p38 (*P*<0.01). The results suggested that minocycline treatment inhibited P2X4R activation and further suppressed the phosphorylation of downstream p38 MAPK.

## 4 Discussion

The present study showed that a big enough dose of minocycline treatment could improve the neurological function score, and reduce cerebral hemorrhage and edema in a rat SAH model. During this process, the P2X4R on microglia and its downstream p38 activation were suppressed by minocycline treatment.

In our study, a total therapeutic dose of minocycline at 100 mg/kg·day (twice daily) could effectively alleviate the symptoms of SAH, but the improvement was limited at a total dose of 50 mg/kg·day. In the studies by Sherchan et al. and Li et al., a single dosage of 135 mg/kg·day was adopted [14, 17]. The study of Guo et al. applied a total dosage of 90 mg/kg·day (twice daily) [13]. If minocycline is to be used in humans, the equivalent dose may reach 8001500 mg/day, much higher than the maximum human dose previously reported [23, 24]. However, in a phase II clinical trial of minocycline in acute spinal cord injury, the patients were well tolerated to a dose of 800 mg/day except one subject displayed elevated liver enzymes [25]. Mice models indicated that a high concentration of minocycline delivered into the central nervous system was required to achieve neurological benefits [26].

Previous studies suggested that minocycline could play a neuroprotective role in SAH through multiple mechanisms. Minocycline could attenuate neuronal loss in the hippocampus and cortex [17]. In the hippocampus, expression of MMP-9 was inhibited by minocycline [13]. Minocycline could also reduce expression of NLRP3 inflammasome and P53-induced apoptosis in the cortex [14]. The anti-inflammatory and anti-apoptotic effects of minocycline reported in those previous studies were also observed in our study. The results of our study suggested a new regulatory mechanism of minocycline as inhibiting the activation of P2X4R on microglia. The most well-known function of P2X4R is to participate in the pain hypersensitivity after peripheral nerve injury [27]. Occurrence of neuropathic pain also involves the activation of microglia and the release of cytokines, excitatory amino acids, ROS, and chemokines, which are closely related to neurological impairment [28]. Current research progress has suggested that P2X4R plays an important role in maintaining normal function of the brain [29, 30, 31]. The involvement of P2X4R is mainly reflected in mediating the secretion of cytokines and chemokines by microglia, regulating clearance of ROS, and inducing calcium influx [22, 32, 33, 34]. In agreement with those previous studies, the results of our study suggested that activation of P2X4R was related with inflammation reaction and neuronal apoptosis.

In conclusion, minocycline showed a neuroprotective role in a rat SAH model and the regulation of P2X4R activation was involved. Minocycline has been used for many years in orthopaedics and dermatology as a broad-spectrum antibiotic. A lot of clinical data on its dose and safety has been accumulated. Developing new uses of minocycline in treating SAH can not only save huge research costs of exploring new drugs, but also shorten the long approval period of clinical trials. However, the current studies only achieved limited benefits in animal models. There are still many concerns before it can be applied to humans, such as the issue of high dose. There is much work to be done to increase the knowledge of SAH pathophysiology and to develop new therapeutic techniques.
